# Altered topological organization of resting-state functional networks in children with infantile spasms

**DOI:** 10.3389/fnins.2022.952940

**Published:** 2022-09-30

**Authors:** Ya Wang, Yongxin Li, Lin Yang, Wenhua Huang

**Affiliations:** ^1^School of Basic Medical Sciences, Engineering Research Center for Translation of Medical 3D Printing Application, Guangdong Provincial Key Laboratory of Digital Medicine and Biomechanics, National Key Discipline of Human Anatomy, Southern Medical University, Guangzhou, China; ^2^Formula-Pattern Research Center, School of Traditional Chinese Medicine, Jinan University, Guangzhou, China; ^3^Department of Anesthesiology, The Fifth Affiliated Hospital of Southern Medical University, Guangzhou, China

**Keywords:** infantile spasm, graph theory, small-world, modularity, functional brain network

## Abstract

Covering neuroimaging evidence has demonstrated that epileptic symptoms are associated with the disrupted topological architecture of the brain network. Infantile spasms (IS) as an age-specific epileptic encephalopathy also showed abnormal structural or functional connectivity in specific brain regions or specific networks. However, little is known about the topological alterations of whole-brain functional networks in patients with IS. To fill this gap, we used the graph theoretical analysis to investigate the topological properties (whole-brain small-world property and modular interaction) in 17 patients with IS and 34 age- and gender-matched healthy controls. The functional networks in both groups showed efficient small-world architecture over the sparsity range from 0.05 to 0.4. While patients with IS showed abnormal global properties characterized by significantly decreased normalized clustering coefficient, normalized path length, small-worldness, local efficiency, and significantly increased global efficiency, implying a shift toward a randomized network. Modular analysis revealed decreased intra-modular connectivity within the default mode network (DMN) and fronto-parietal network but increased inter-modular connectivity between the cingulo-opercular network and occipital network. Moreover, the decreased intra-modular connectivity in DMN was significantly negatively correlated with seizure frequency. The inter-modular connectivity between the cingulo-opercular and occipital network also showed a significant correlation with epilepsy frequency. Together, the current study revealed the disrupted topological organization of the whole-brain functional network, which greatly advances our understanding of neuronal architecture in IS and may contribute to predict the prognosis of IS as disease biomarkers.

## Introduction

Multiple studies have found that epilepsy is a systemic disorder with disrupted brain networks, rather than a single source of pathophysiology in the human brain (Bernhardt et al., 2013; Vaessen et al., 2013). Infantile spasms (IS), one kind of epileptic encephalopathy, are characterized by clinical spasms and hypsarrhythmia on electroencephalogram (EEG), as well as delayed brain development or regression (Pavone et al., 2014; D’Alonzo et al., 2018). Although, currently, IS may cease by applying antiepileptic drugs, or spontaneously, a majority of IS children are left with other seizure types and motor delay, and often neurocognitive troubles. Considerable efforts have been made in the past decade, but the neural mechanism of IS remains largely unclear.

With the development of neuroimaging methods, understanding the mechanisms of IS has become a research priority currently. Functional magnetic resonance imaging (fMRI) provides an efficient and non-invasive way to explore the functional properties of the human brain (Bullmore, 2012; Goodman and Szaflarski, 2021). Our previous studies had shown that the seizure of IS was related to the impairment of the single functional network called default mode network (DMN) through regional homogeneity and functional connectivity analysis (Tan et al., 2016; Wang et al., 2017). Altered structural remodeling in the temporal lobe was also confirmed in patients with IS with voxel-based morphometry and tract-based spatial statistics (Fosi et al., 2015). Another study also evaluated initial MRI abnormalities with T2-weighted images in cerebral structure in IS children (Harini et al., 2018). Taken together, this neuroimage evidence highlights the abnormal functional interconnections and structural alternations of the brain in IS. The broadly distributed brain changes can be understood by the view of the brain network. The whole brain can be considered as a complex network to integrate various information inputs across multiple-distributed systems (He and Evans, 2010; Barch, 2013). In epilepsy, the seizure spreads across the cortical surface based on the network pathways. Broadly, changes in brain activation and structure would be detected. Thus, the network approach is an important way to explore the neural mechanism of epilepsy. The frequently used network approach is a seed-based functional connectivity. Although this approach can provide information about how regions are related to each other, it cannot reveal whole-brain connectivity patterns and topological organization. Complex network analysis is a powerful tool to map and characterize the connectivity patterns of the brain. A complex network analysis of IS can provide a complete view of how the disease affects children’s brains. Currently, few published articles have focused on the topological organization of the whole-brain functional networks in patients with IS.

Many complex networks, such as social network, own efficient and economical topology to support both segregated and integrated information transmission. The characteristics and methods in graph theory that describe network topological properties employed in the analysis of brain networks came from neuroimaging techniques (e.g., MR images, EEG, and magnetoencephalography) (Bassett and Bullmore, 2006). The human brain networks are confirmed to exist with optimized topological properties, such as the small-world principle, high transmission efficiency, and modularity structure using these methods (Bullmore and Sporns, 2009; Bullmore and Sporns, 2012). In addition, the alterations of these properties have been detected in various clinical disorders, such as attention deficit hyperactivity disorder (Chen et al., 2019), depressive disorder (Sheng et al., 2022), Alzheimer’s disease (Kim et al., 2015), stroke (Li Y. et al., 2021), epilepsy (Li R. et al., 2021), and schizophrenia (Hadley et al., 2016). Recently, our group also used the graph theory method in children with generalized tonic-clonic seizures and demonstrated disrupted topological organizations of their functional and structural networks in epileptic children (Li et al., 2020a,b). They all together suggest that graph theory-based network analysis could reveal the underlying system-level changes of different processes, which promote our understanding of the physiological mechanism in some respects. Paldino et al. (2017) predicted focal epilepsy duration based on global features of resting-state fMRI by machine learning algorithm and confirmed that the global brain network metrics, including the modularity, path length, and global efficiency, were independently related to the epilepsy duration. Meanwhile, a previous study also found abnormal small-world metrics and network efficiency. For example, the functional brain network in focal epilepsy displayed decreased path length and small-worldness (Park et al., 2018) and the temporal lobe epilepsy also exhibits abnormal small-world properties (Liao et al., 2010). In addition, the modular organization based on the fMRI brain network also shows topological changes in different epilepsies (Liao et al., 2010; Vaessen et al., 2013, 2014; Xu et al., 2013). For example, the brain network in mesial temporal lobe epilepsy demonstrated decreased connectivity within the parietal and frontal lobes (Liao et al., 2010), while the brain network in frontal lobe epilepsy showed different connected patterns with increased inner-modular and decreased inter-modular connectivity (Vaessen et al., 2014). These above global network metrics with inconsistent changes could help define epilepsy-related markers. Therefore, in this study, we employed small-world topology and modularity to explore the topological changes of seizure to the functional brain’s intrinsic activity in patients with IS.

Here, we hypothesized that patients with IS may undergo disruptions in the small-world properties and intra- and inter-modular connectivity. To test our hypothesis, we leveraged resting-state functional MR images, which were often used to measure intrinsic or spontaneous brain neuronal activity to construct the whole-brain functional connectome (Fox and Raichle, 2007). Then, by calculating the topological metrics with graph theoretical approaches, we attempt to find whether the seizure disrupts the whole-brain topological architecture of functional networks in patients with IS. If so, whether the altered topological properties are associated with clinical characteristics?

## Materials and methods

### Subjects

A total of 51 right-handed subjects were recruited from the Shenzhen Children’s Hospital, including 17 patients with IS (5 girls; mean 2.5 years) and 34 age- and sex-matched healthy control participants (13 girls; mean 2.5 years). The data of one patient had been removed because of excessive head motion. All patients underwent comprehensive clinical assessments, including detailed seizure history and video-EEG telemetry (23 channels), and met the following inclusion criteria: (1) typical clinical symptoms of IS, such as spasms, hypsarrhythmia, and impaired consciousness; (2) evident EEG findings and at least one seizure and consistent with the diagnosis of IS; and (3) without accompanying neurological or psychiatric disorders other than epilepsy. All the subjects accepted resting-state functional MRI scanning at the time of recruitment. Written informed consents were obtained from the parents/legal guardians of all enrolled children. This study was approved by the Ethics Committee of Shenzhen Children’s Hospital, and the method was carried out in accordance with the approved guidelines.

### Image acquisition

All functional MR images were performed on a 3T scanner (MAGNETOM Trio Tim, Siemens, Germany) with an eight-channel head coil at the Shenzhen Children’s Hospital, Guangdong, China. We obtained the data using an echo planar imaging sequence with parameters: TR/TE = 2,000/30 ms, matrix = 94 × 94, flip angle = 90°, FOV = 220 mm × 220 mm, slice thickness = 3 mm, 36 interleaved axial slices, and 130 volumes. During the entire scanning procedure, all the subjects under 4 years were sedated with 10% choral hydrate (dosage: 50 mg/kg/time, the maximum dose was 1 g). In the IS group, four subjects (24%) were not sedated compared with seven subjects (21%) who were not sedated in the control group. Others were instructed to keep their eyes closed and relax their minds without falling asleep. To avoid these influences, we performed a relatively short scan, observed the whole process, and asked about their conditions after scanning. A foam cushion and headphones were used to minimize head motion and scanner noise.

### Data processing

The resting-state functional MR image preprocessing was performed by the GRETNA toolbox^1^, which is based on the Statistical Parametric Mapping (SPM8^2^) (Wang et al., 2015). First, to ensure magnetization equilibrium, we removed the first 10 volumes. Then, we performed slice timing correction and realignment aiming at the remaining volumes. One patient was excluded from further calculations based on the criterion of head motion >2 mm and/or rotations >2°. The corrected data were then normalized to the Montreal Neurological Institute (MNI) space by estimating their transformation to the echo-planar imaging (EPI) template (Ashburner and Friston, 1999) and resampled into a voxel size of 3 mm × 3 mm × 3 mm thereafter. The resulting images were spatial smoothing by convolution with an isotropic Gaussian kernel (FWHM = 6 mm), then temporally band-pass filtered (0.01–0.08 Hz) to minimize the effects of low-frequency drift and high-frequency physiological noise, such as respiratory and cardiac noise. Linear trends were also removed. The 24 head motion parameters (Yan et al., 2013), global signal (Birn, 2012; Power et al., 2014; Murphy and Fox, 2017; Khatri and Kwon, 2022), mean white matter (WM) signal, and cerebrospinal fluid (CSF) signal were also regressed out from each voxel’s time course. Finally, image volumes with Framewise Displacement (FD) >0.5 were scrubbed by replacing the frames of poor quality with linear interpolation to reduce the effects of head motion (Power et al., 2012).

### Functional connectivity matrix construction

In this section, we constructed individual inter-regional functional connectivity matrices, of which the network nodes stand for brain regions of interest (ROIs) and edges between nodes stand for functional associations among different regions of the brain. The whole brain was parcellated into 160 ROIs functionally to define the network nodes, and then the mean time series for each region was extracted. We chose Dosenbach’s 160-ROIs parcelation as our scheme since it broadly covers both the cerebral cortex and cerebellum, and also these functionally defining ROIs were obtained through meta-analysis based on large fMRI activation data, which thus could provide the additional foundation for interpreting developmental changes (Dosenbach et al., 2010). The pairwise functional association was estimated among the time series by computing Pearson correlation coefficients. We also performed Fisher’s *r*-to-*z* transformation to improve the normality of the correlations. For network topology, we chose positive correlations to minimize effects on test–retest reliability. To exclude the confounding effects of spurious relationships in functional matrices, we applied a sparsity threshold to ensure the same number of edges for each matrix by applying a subject-specific connectivity strength threshold, and therefore, permitting an examination for relative network organization (He et al., 2009a). We set the sparsity threshold at multiple densities ranging from 0.05 to 0.4 at an interval of 0.02 based on previous experience to obtain a more efficient binary network than a random network.

### Network metrics

In the study, we computed the properties of the functional networks with routines from the GRETNA toolbox to reflect the topological alterations in patients with IS. The network topological properties at the global level were collected, including clustering coefficient (*C*_*p*_), characteristic path length (*L*_*p*_), local efficiency (E_*loc*_), global efficiency (E_*glob*_), and modularity.

Small-world as an attractive architecture for the description of complex brain networks could reflect both specialized and integrated information processing and maximize the efficiency in information transmission at a low-wiring cost (Watts and Strogatz, 1998; Bassett and Bullmore, 2006; Li et al., 2014). The small-world topology is characterized through *C*_*p*_ and L_*p*_, which quantifies the extent of local interconnectivity or cliquishness and the extent of overall communication efficiency of a network separately and could imply the segregation and integration of the network, respectively (Watts and Strogatz, 1998; Sporns and Zwi, 2004; Rubinov and Sporns, 2010). We benchmarked these above metrics against the random graph to evaluate the topological metrics of the brain networks (Maslov and Sneppen, 2002). Thus, in this study, the normalized clustering coefficient (γ), normalized path length (λ), and the small-worldness (σ = γ/λ) were leveraged to evaluate the small-world measures of the functional networks. Of note, the network that meets the criteria (γ > 1 and λ ≈ 1 or σ = γ/λ > 1) was recognized as existing small-world properties, which thus ensures efficiently transmits information at both the local and global levels within this network.

Efficiency stands for the ability to exchange parallel information with low consumption and could be described at global and local levels (Latora and Marchiori, 2001). Meanwhile, the human brain functional network exist with economical small-world properties and could support the high-efficient transfer of parallel information (Achard and Bullmore, 2007). Therefore, we leverage the E_*loc*_ and E_*glob*_ to measure the ability to transmit information at the global and local levels separately (Latora and Marchiori, 2001).

Modules mean to a bunch of nodes joining together in closely integrated groups between which there are only sparser connections in a network (Newman, 2006). Here, modularity could contribute to identify groups of functionally associated components that possess specific biological functions. In this study, we adopted the parcelation schemes of Dos-160, which divides the whole brain into six sub-networks (e.g., cingulo-opercular network, fronto-parietal network, default network, sensorimotor network, occipital network, and cerebellum network) (Dosenbach et al., 2010). We then performed modularity analysis with spectral optimization in the undirected graphs following this parcelation. After that, we calculated the averaged functional connectivity strength within and between the six modules. Finally, we calculated the area under the curve (AUC) of the network measures, which provided us with a summarized scalar for the topological characterization of the brain networks independent of single threshold selection. The AUC has been confirmed to be sensitive to detecting topological alterations of brain disorders by previous studies (He et al., 2009a; Wang et al., 2009).

In addition, to confirm the result’s robustness of the graph theory method, we also performed another parcelation scheme, which parcellates the whole brain into 264 ROIs functionally (Power et al., 2011).

### Statistical analysis

#### Differences in network metrics

To determine whether there existed significant group differences in the network properties, we performed statistical comparisons with a two-sample t-test with the AUC, including λ, γ, σ, E_*glob*_, and E_*loc*_ between the two groups (*p* < 0.05, FDR corrected). In addition, we also compared the AUC of the averaged functional connectivity strength over the sparsity from 0.05 to 0.4 within and between the modules (*p* < 0.05, FDR corrected). We performed all the above comparisons with GRETNA. Age and gender as additional covariates were regressed in all the analyses above. Moreover, the Hedges’ *g* was used to calculate the effect size and quantify the difference in network metrics between the two groups with a relatively small sample size (Hentschke and Stüttgen, 2011). The magnitude of Hedges’ *g* refers to the criteria of Cohen’s *D*: *d* (0.01–0.19) = very small, *d* (0.2–0.49) = small, *d* (0.5–0.79) = medium, *d* (0.8–1.19) = large, *d* (1.2–1.99) = very large, and *d* (≥ = 2) = huge (Sawilowsky, 2009).

#### Association of network measures and clinical characteristics

To detect whether the patient’s clinical characteristics had a mutual influence on brain topological changes, Pearson correlation between the network metrics with significant differences and the epilepsy duration and frequency were calculated. We performed a partial correlation analysis to remove the influence of age and sex. We used a significance level of *p* < 0.05, uncorrected.

## Results

### The basic demographical and clinical characteristics of subjects

A total of 51 right-handed subjects were recruited, which include 17 patients with IS and 34 age- and sex-matched healthy control participants. Pearson’s Chi-square test showed no significant difference in gender (χ^2^ = 0.386, *p* = 0.534) and two sample *t*-tests showed no significant difference in age (*t* = 0.016, *p* = 0.988) between the two groups. Specific demographic and clinical information of all patients with IS is provided in Table 1.

**TABLE 1:** Characteristics of the IS patients.

Patient	Sex	Age (month)	Onset time (month)	Type	Pathogeny	Seizure frequency (time/day)	Antiepileptic drugs
1	F	23	4	No lesion	No	3–4	LEV
2	M	83	24	No lesion	No	10–20	VPA TPM
3	M	8	0.5	Lesion	Temporal lobe cortex dysplasia	1–2	LEV VPA
4	M	18	6	Lesion	Cerebromalacia of right hemisphere	3–15	TPM
5	M	20	13	Lesion	Cerebromalacia of left hemisphere and right temporal lobe	10–20	VPA OXC
6	M	82	24	No lesion	Bilateral gray matter heterotopia	1–2^a^	LEV TPM
7	M	80	6	Lesion	Cerebromalacia of left hemisphere	3–4	CBZ VPA
8	F	8	4	Lesion	Left hemisphere cleft deformity	2–3	CBZ
9	M	12	1	No lesion	No	5–10	VPA LEV
10	M	14		No lesion	Ventricular expansion		Ketogenic diet
11	F	13	5	No lesion	Bilateral parietal-occipital cortex dysplasia	7–10	TPM LEV
12	M	71	3	No lesion	Signal of right anterior cingulate cortex abnormal	1–2	TPM LEV
13	M	42	9	Lesion	Signal of right mesial temporal lobe abnormal	1–2	LEV OXC
14	M	5	5	No lesion	Bilateral pachygyria deformity	1^b^	TPM OXC
15	M	7	7	Lesion	Right temporal lobe lesion	1–3^c^	LEV
16	F	5	4	No lesion	No	4–5	LEV TPM
17	F	25	12	Lesion	Atrophy of the left hemisphere	10^a^	TPM OXC

^a^Time/year; ^b^time/month; ^c^time/week.

LEV, levetiracetam; VPA, valproic acid; TPM, topiramate; OXC, oxcarbazepine; CBZ, carbamazepine.

### Changes in brain functional network topological metrics

**FIGURE 1:**
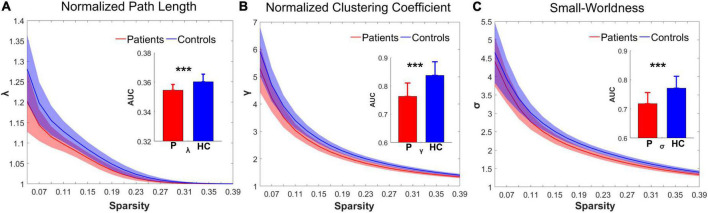
Group differences in the small-world topological metrics between the IS and control groups. In the range of sparsity (0.05–0.4), the topologies of **(A)** λ, **(B)** γ, and **(C)** σ in both groups exhibited small-world property. Bar charts plot the significant differences of the AUC of λ, γ, and σ between the IS children and controls (*p* < 0.05, FDR corrected). ***Significant difference between the two groups. P, patient group; HC, healthy control group.

**FIGURE 2:**
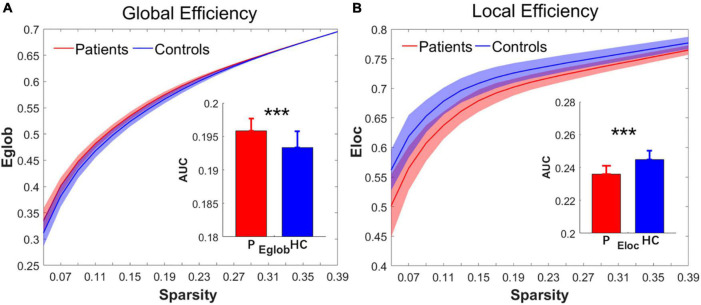
Group differences in the network efficiency of **(A)** E_glob_ and **(B)** Eloc between the IS and control groups in the range of sparsity (0.05–0.4). Bar charts plot the significant differences of the AUC of E_glob_ and E_loc_ between the IS children and controls (*p* < 0.05, FDR corrected). ***Significant difference between the two groups. P, patient group; HC, healthy control group.

Over the sparsity range from 0.05 to 0.4 (step = 0.02), both the IS group and the control group exhibited high-efficiency small-world topology. In the premise of common small-world architecture, it appeared significant differences in the small-world parameters and network efficiency between the two groups. Compared with the controls, the IS group showed significantly decreased AUC of λ (*t* = −4.268, *p* = 0.000), γ (*t* = −5.269, *p* = 0.000), and σ (*t* = −4.564, *p* = 0.000) after FDR correction. The results are shown in Figures 1A–C. Regarding network efficiency, the AUC comparisons revealed a significantly increased E_*glob*_ (*t* = 4.009, *p* = 0.000) and significantly decreased E_*loc*_ (*t* = −5.993, *p* = 0.000) after FDR correction in the functional networks of patients with IS. The results of the network metrics are shown in Figures 2A,B. The λ and Eglob showed large effect sizes between the two groups (λ: Hedges’ *g* = −1.185, 95% CI = −1.8125 to -0.558; Eglob: Hedges’ *g* = 1.117, 95% CI = 0.495–1.740). The γ, σ, and Eloc exhibited very large effect sizes between the IS and control groups (γ: Hedges’ *g* = −1.435, 95% CI = −2.082 to -0.788; σ: Hedges’ *g* = −1.270, 95% CI = −1.903 to -0.636; Eloc: Hedges’ *g* = −1.610, 95% CI = −2.272 to -0.946).

**FIGURE 3:**
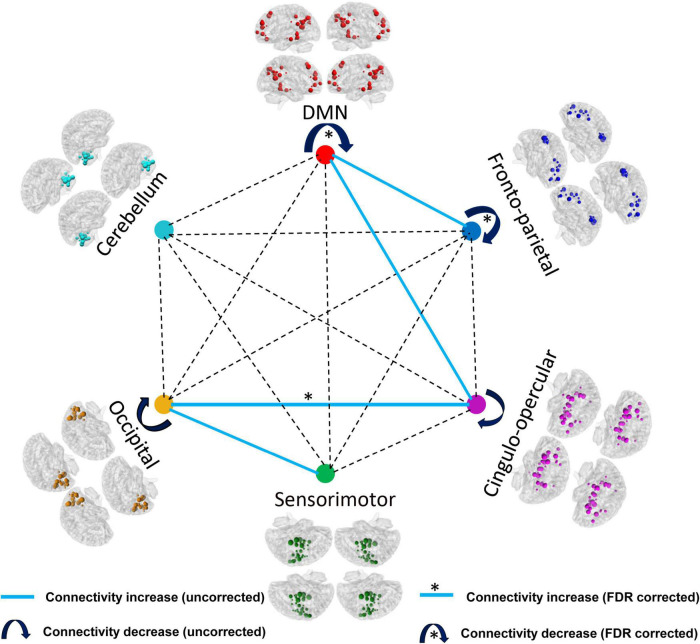
Comparison results of the AUC of the averaged functional connectivity strength within and between the modules (*p* < 0.05, FDR corrected). *The connectivity can withstand the FDR correction.

According to the modular architecture of the Dos-160 template, we found a significant reduction of the averaged functional connectivity strength within DMN (very large effect size), fronto-parietal network (large effect size), and significant enhancement of the averaged functional connectivity strength between occipital network and cingulo-opercular network (large effect size) after FDR correction. In addition, we found that the intra-modular connection decreased (medium effect size), such as the cingulo-opercular network and occipital network. On the contrary, the inter-modular connection mostly increased (medium effect size), such as DMN and fronto-parietal network, DMN and cingulo-opercular network, sensorimotor network, and occipital network without correction. The details of the results of the modules are shown in Table 2 and Figure 3.

**TABLE 2:** The altered edge numbers of connection within or between modules.

Modules	*p*	Correction	Effect size (Hedges’ *g*)	Edge numbers of connection
**Intra-modular connections**				
DMN	0.000	FDR (0.004)	−1.207^a^	↓
Fronto-parietal network	0.001	FDR (0.013)	−1.026^b^	↓
Cingulo-opercular network	0.016	Uncorrected	−0.753^c^	↓
Occipital network	0.012	Uncorrected	−0.780^c^	↓
**Inter-modular connections**				
DMN and fronto-parietal network	0.047	Uncorrected	0.611^c^	↑
DMN and cingulo-opercular network	0.021	Uncorrected	0.675^c^	↑
Occipital and cingulo-opercular network	0.004	FDR (0.027)	0.894^b^	↑
Sensorimotor and occipital network	0.019	Uncorrected	0.730^c^	↑

^a^Very large; ^b^large; ^c^medium effect sizes.

The results using 264 ROIs showed a similar trend with significantly decreased AUC of the λ, γ, σ (Supplementary Figure 1), Eloc, and significantly increased E_*glob*_ (Supplementary Figure 2) after FDR correction. We also found a significant decline of the averaged functional connectivity strength within DMN, fronto-parietal task control network, and significant ascension of the averaged functional connectivity strength between DMN and module 3 (salience, cingulo-opercular, etc.) after FDR correction, which is consistent with the results above. The details including the *p*-values and effect sizes are shown in Supplementary Table 1.

### Association of network properties and clinical variables

**FIGURE 4:**
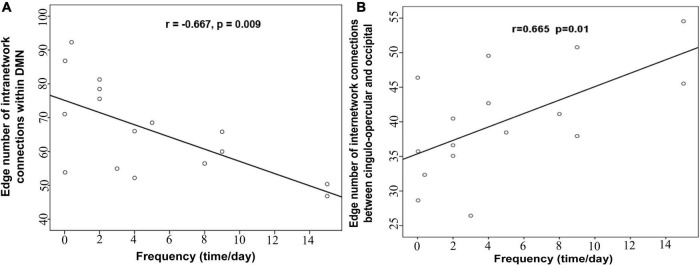
The correlation **(A)** between the intra-modular connections within DMN and the epilepsy frequency, **(B)** between the inter-modular connections and the epilepsy frequency.

We analyzed the correlations between clinical characteristics (such as epilepsy duration and frequency) and network properties (altered global topological parameters and edge number of modular connections) in patients with IS. Of note, the epilepsy frequency of one IS patient is missing. Partial correlation analysis showed that intra-modular connectivity within DMN was significantly negatively correlated with epilepsy frequency after controlling confounding variables (*r* = −0.667, *p* = 0.009; Figure 4A). The inter-modular connectivity between the cingulo-opercular and occipital network also showed a significant correlation with epilepsy frequency (*r* = 0.665, *p* = 0.01; Figure 4B). However, no remarkable correlation was found between the global topological metrics and clinical variables.

According to the results of 264 ROIs, we also found a significant negatively correlation between the connectivity within DMN and epilepsy frequency (*r* = −0.746, *p* = 0.002; Supplementary Figure 3).

## Discussion

This is the first study to investigate the topological organization of functional networks in patients with IS with graph theoretical approaches according to brain functional MRI. In this study, we analyzed the alterations of small-world topology and modularity of the resting-state functional network in patients with IS and detected the correlation between the altered metrics and clinical properties. The results can be summarized as follows: (1) patients with IS showed a significant decrease in the small-worldness index and significant changes in their global and local network efficiency compared with the normal controls; (2) significantly disrupted intra-module integration within DMN and fronto-parietal network and enhanced inter-module averaged functional connectivity strength between occipital network and cingulo-opercular network. (3) The altered connectivity strength within DMN was negatively correlated with the frequency of seizures, and the inter-modular connectivity between the occipital network and the cingulo-opercular network was positively correlated with the frequency of seizures in patients with IS. Taken together, our study provides strong evidence for the disrupting of topological organization in the functional network in IS based on two parcelation schemes. These findings may greatly deepen our understanding of the topological mechanism underlying the spontaneous brain intrinsic activity in IS.

### Changes in small-world topology in patients with infantile spasms

According to the mathematical verdict, a network exists in small-world when it meets the following two conditions: γ > 1 and λ ≈ 1. Previous studies involving complex brain networks also confirmed the existence of small-world topology in both healthy and diseased states (Bullmore and Sporns, 2012; Sporns, 2013; Wen et al., 2017; Li et al., 2018; Chen et al., 2019). In our study, both the IS and the control group also exhibited robust small-world properties. Small-world properties mean that the human brain showed an optimal architecture and the brain network has high efficiency in information transmission (Wang et al., 2010). Our recent work on children with generalized epilepsy has also proven the small-world property of the brain’s functional and structural network (Li et al., 2020a,b).

Despite the common small-world topology, patients with IS showed decreased λ, γ, σ, E_loc_, and increased E_glob_. The short λ and high E_glob_ express a great ability to integrate global functional information dissemination in the larger and sparser network (Estrada and Hatano, 2008; Rubinov and Sporns, 2010). The IS-related changes in λ and E_glob_ could contribute to increased long-distance functional connections, which may enhance whole brain propagation of information flow. A previous study about small world neuronal network considered that phase synchronization as a function of the locality of network connections changes from local coherence to global coherence dependent on the distance between two neurons (Percha et al., 2005). This may contribute to our comprehension. The high γ and E_loc_ exhibit the ability for specialized processing to occur within densely interconnected groups of brain areas. In the present study, the IS-related decreases in γ and E_loc_ indicate a relatively sparse interconnection between local brain regions and decreased capacity to pass information within the neighbors, suggesting a possible decline in the separation function of brain cognitive processing. A study in epileptic patients with different handle methods also exhibited the same alterations in network efficiency (Song et al., 2015). One recent research in epilepsy also found a significant decrease in E_loc_ and an increase in E_glob_ in children with generalized tonic-clonic seizures (Li et al., 2020a). The human brain functional network may through increasing the global efficiency compensate for the decreased efficiency of local regions in patients with IS. The lower σ reflects the disrupted proportion of integration and differentiation in the brain network in patients with IS.

To sum up, the current results show that the topological architecture of the global brain functional networks were disrupted in IS characterized by the reduced capacity of information dissemination between local regions and higher whole brain propagation of information flow. The current results may reflect the imbalance between functional segregation and integration in the brain networks of patients with IS and a tendency toward randomization to some extent.

### Changes in modularity in patients with infantile spasms

Modular structure as an important organizational principle of complex biological networks has been widely studied in many other networks recently (Girvan and Newman, 2002; Guimera et al., 2005). Nowadays, increasing studies employ this property of modularity to study human brain networks and the results support the existence of the modular architecture in brain functional networks (Newman, 2006; He et al., 2009b; Meunier et al., 2010; Gallen and D’Esposito, 2019). Compared to global topological properties, the detection and characterization of modular architecture could contribute to materialize the groups of functionally and/or anatomically associated components, which are related with specific biological functions, and detect the alterations within and between them (Wang et al., 2010). In this study, we applied the modular parcelation schemes of Dos-160 to reduce the disturbance on account of fewer cases to detect the changes in local topological architecture within and between the six modules.

According to the results, there are significant differences in functional connectivity at the intermediate modular level between the healthy controls and the patients with IS. The number of connections within DMN and the fronto-parietal network was significantly reduced. In addition, the connections within the cingulo-opercular network and occipital network also decreased in spite of being non-significant. However, the functional connectivity strength between DMN and fronto-parietal network, DMN and cingulo-opercular network showed an increasing trend. The functional connectivity strength between the cingulo-opercular network and the occipital also showed a significant increase. DMN is considered to be related to a diverse series of functions including episodic memory, self-referential mental processing, and supervising the external environment (Buckner et al., 2008). In the brains in children with epilepsy, the abnormal internal activities in DMN may underlie poor brain development or regression. In the present study, the decreased connectivity strength in DMN may reflect disrupted neuronal activity within the DMN regions. This result is consistent with our previous study, which showed significantly reduced functional connectivity and lower low-frequency fluctuation in DMN regions in patients with IS (Wang et al., 2017). Other types of epilepsy with mental disorders also showed decreased integration within DMN even during resting interictal durations without interictal epileptiform discharges (Gonen et al., 2020; Parsons et al., 2020; Yang et al., 2021). Our previous studies in children with generalized tonic-clonic seizures also found significant decrease in betweenness centrality and functional connectivity of the DMN regions (Li et al., 2020a,b). The functional damage of DMN in these previous studies and our present results can explain the neuroimaging expression for the damage of cognitive function and the reduction in functional integrations of the DMN in children with epilepsy. In addition, our results also showed a significant negative correlation between the functional connectivity strength and epileptic frequency. This result further indicates that the epileptiform discharges in patients with IS may lead to and aggravate the disruption of neuronal activity in DMN regions, which triggers the reduction in blood oxygenation level-dependent on the increase of epileptic frequency (Shmuel et al., 2006). In this study, the decreased connections were mainly located within DMN and fronto-parietal network, while increased connections were mainly located between DMN and fronto-parietal network or cingulo-opercular network. The increased connections may represent a compensatory mechanism for the disrupted neuronal activity within DMN or the fronto-parietal network to transmit functional information. Enhanced inter-modular connections and decreased intra-modular connections indicated that the architecture of the brain networks was reconfigured in children with IS. Thus, our modular analysis results provide further evidence for the functional disruption of the whole-brain system in patients with IS.

The subcortical network, cingulo-opercular network, also showed increased functional connectivity strength with cortical network DMN and occipital network in children with IS. In addition, the functional connectivity strength between the cingulo-opercular network and the occipital network was significantly correlated with epileptic frequency. This result indicates that the clinical seizure in patients with IS may contribute to the information transmission between cortical regions and subcortical nuclei. A previous study about the neuronal network with electroencephalogram considered that slow wave activity within the hypsarrhythmia in patients with IS was correlated with blood oxygenation level-dependent signal in brainstem, cortex, and subcortical structures (Siniatchkin et al., 2007). Another study with source analysis in West syndrome also supports the theory that hypsarrhythmia results from ascending brainstem pathways that project widely to subcortical nuclei and cerebral cortex (Japaridze et al., 2013). This theory contributes to our understanding of the underlying increased functional connectivity strength between cortical regions and subcortical nuclei. While the decreased functional connectivity strength within the cortex in our study might be compensated by increased connectivity between cortical regions and subcortical nuclei. Further study about the alterations in cerebral cortex structures would be performed.

### Limitations

Several limitations need to be further addressed. First, some participants were sedated with 10% chloral hydrate during the neuroimaging scanning, which may affect the analysis results of the network topological metrics. We thus compared the two groups in which all the subjects without sedation were excluded. Even though we discovered similar global topological changes (Supplementary Figure 4), further comparison between the subjects with and without sedation in a large sample size will strengthen our conclusion. Second, the fMRI data length of 130 volumes is relatively short, which might affect the results to some extent. Longer scan time or scanning twice or more times at short time may strengthen the credibility of our results. Third, a larger sample size is needed to contribute to the stability of the results. In addition, combining multimodal neuroimaging data may help to clarify the pathological mechanism and uncover structure-function relationships in patients with IS.

## Conclusion

In the current study, we employed graph theoretical analysis to detect the reorganization of brain functional topological architecture at the whole-brain level and the functional network level. The results demonstrate that the global properties and the modular structure were disrupted in patients with IS compared with healthy controls. The decreased γ and E_loc_ reflect the disrupted capacity of information transmission at the global level. At the same time, the decrease of functional connectivity strength within DMN, fronto-parietal network, cingulo-opercular network, and occipital network also support the alterations of global properties. In addition, the increased functional connectivity between modules also showed the same tendency with increased E_glob_. Our findings suggest that the topological organization is disrupted in patients with IS. These properties may serve as indicators for us to understand the pathogenesis in patients with IS.

## Data availability statement

The raw data supporting the conclusions of this article will be made available by the authors, without undue reservation.

## Ethics statement

The studies involving human participants were reviewed and approved by the Ethics Committee of Shenzhen Children’s Hospital. Written informed consent to participate in this study was provided by the participants’ legal guardian/next of kin.

## Author contributions

YW: software, writing—original draft preparation and reviewing and editing. YL: methodology, funding acquisition, and data curation. LY: writing—reviewing and editing. WH: conceptualization and supervision. All authors contributed to the article and approved the submitted version.

## Footnotes

1
http://www.nitrc.org/projects/gretna/

2
http://www.fil.ion.ucl.ac.uk/spm/
