# Supplementary material for: Altered topological organization of resting-state functional networks in children with infantile spasms
Source: Front Neurosci. 2022 Sep 30;16:952940. doi: 10.3389/fnins.2022.952940 (PMC9562010; doi:10.3389/fnins.2022.952940)
Supplement: Supplementary file 1 [file Data_Sheet_1.docx]

***Supplementary Material***

1. **Supplementary Figures and Tables**
   1. **
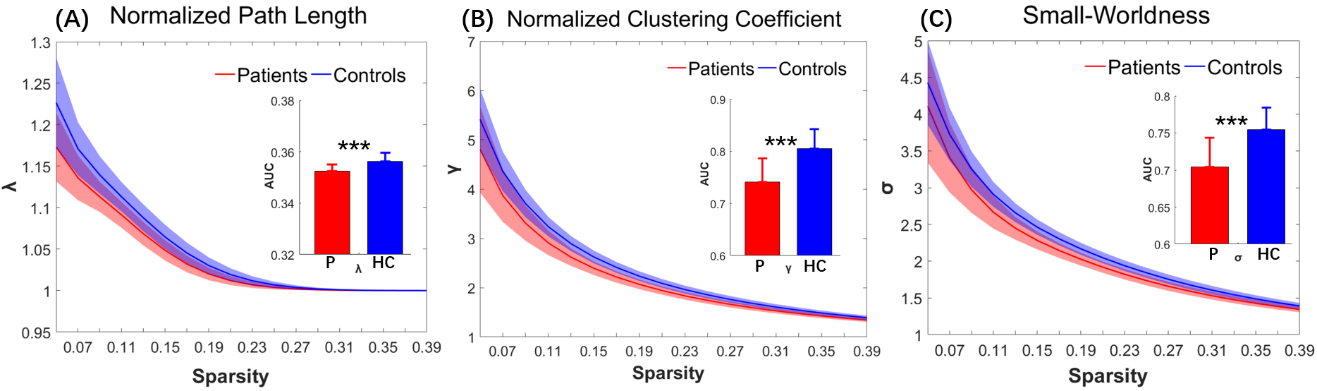
Supplementary Figures**

**Supplementary Figure 1.** Group differences in the small-world topology between the patient and control group based on 264 ROIs. In the range of sparsity (0.05~0.4), the topologies of (A) λ, (B) γ, and (C) σ in both groups exhibited small-world property. Bar charts plot the significant differences of the AUC of λ (t =-4.141, *p* =0.000), γ (t =-5.855, *p* =0.000), and σ (t =-5.299, *p* =0.000) between the IS children and controls after FDR correction. The above three metrics also showed large/very large effect sizes between the two groups (λ: Hedges’ g = -1.176, 95% CI =-1.802 to -0.549; γ: Hedges’ g =-1.589, 95% CI =-2.250 to -0.927; σ: Hedges’ g =-1.454, 95% CI =-2.103 to -0.805).

*** Indicates a significant difference between the two groups.

P = patient group, HC = healthy control group.

**
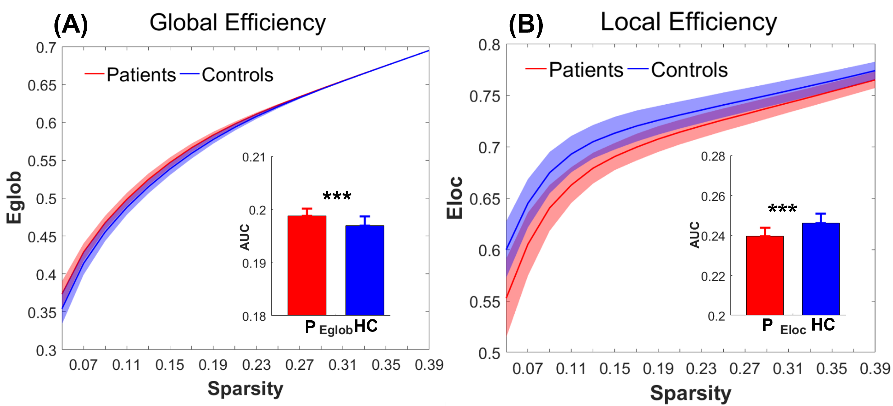
**

**Supplementary Figure 2.** Group differences in the network efficiency of (A) E_glob_ and (B) E_loc_ between the patient and control group in the range of sparsity (0.05~0.4) based on 264 ROIs. Bar charts plot the significant differences of the AUC of E_glob_ (t =3.867, *p* =0.000) and E_loc_ (t =-5.579, *p* =0.000) between the IS children and controls after FDR correction. The above two metrics also showed large/very large effect sizes between the two groups (Eglob: Hedges’ g =1.108, 95% CI =0.486 to 1.730; Eloc: Hedges’ g =-1.504, 95% CI =-2.157 to -0.851).

*** Indicates a significant difference between the two groups.

P = patient group, HC = healthy control group.

**
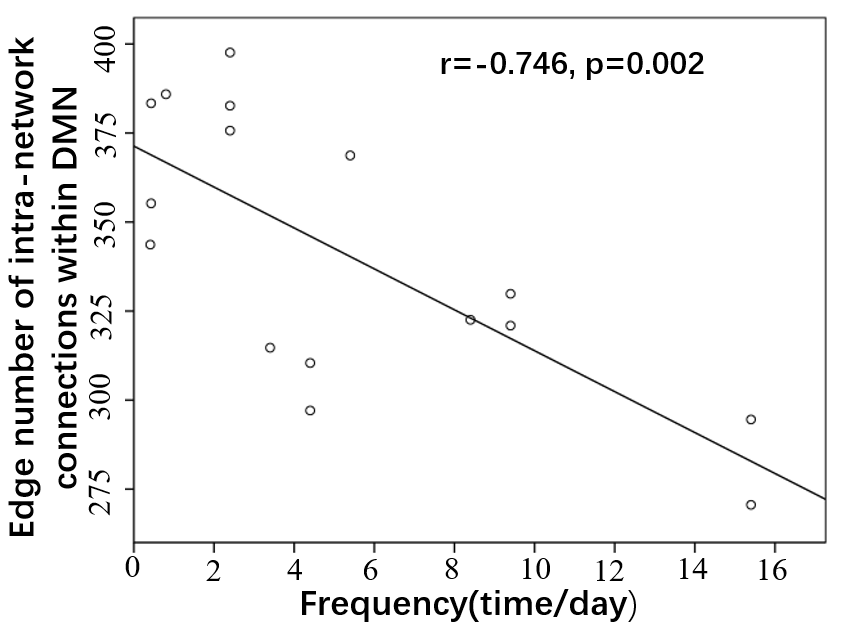
**

**Supplementary Figure 3.** The correlation between the intra-network connections within DMN and the epilepsy frequency.

**
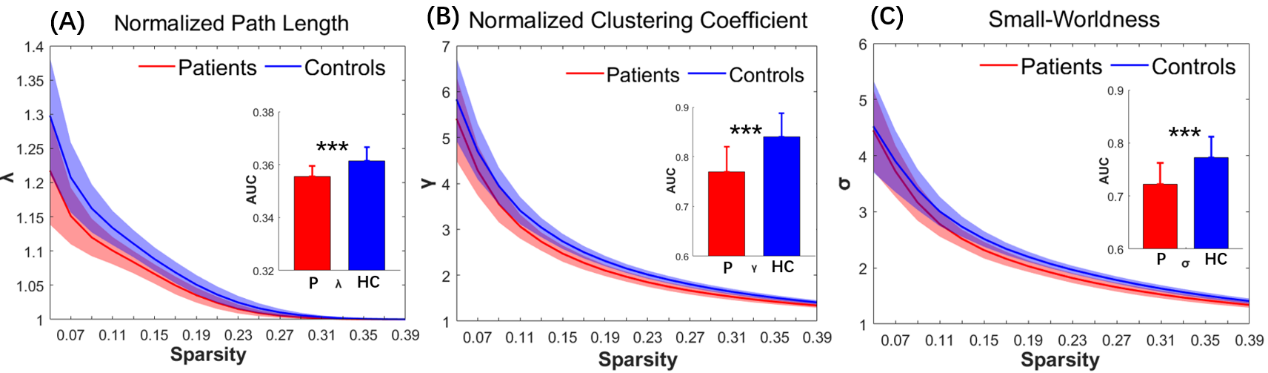
**

**
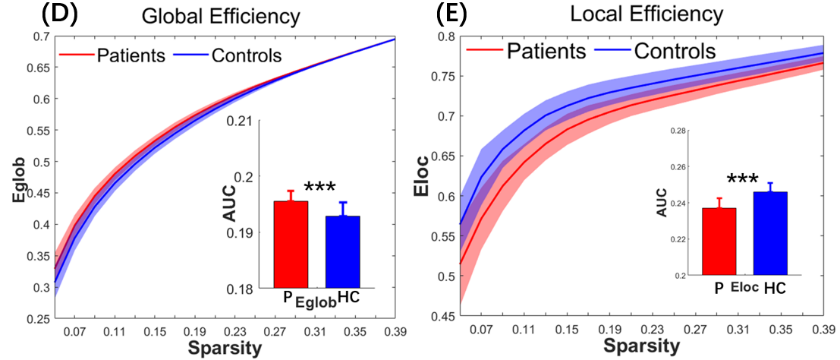
**

**Supplementary Figure 4.** Group differences in the global topological metrics between the IS (13 subjects) and control group (27 subjects) in the range of sparsity (0.05~0.4) based on 160 ROIs. The subjects without sedation were excluded. Bar charts plot the significant differences with significantly decreased AUC of the λ (p = 0.001, t =-3.554), γ (p = 0.000, t =-4.447), σ (p = 0.001, t =-3.864), Eloc (p = 0.000, t =-4.927), and significantly increased Eglob (p = 0.002, t =3.312) after FDR correction. In addition, large/very large effect sizes were showed on all the features between the two groups (λ: Hedges’ g =-1.255, 95% CI =-1.974 to -0.536; γ: Hedges’ g =-1.464, 95% CI =-2.202 to -0.725; σ: Hedges’ g =-1.258, 95% CI =-1.977 to -0.539; Eglob: Hedges’ g =1.168, 95% CI =0.457 to 1.880; Eloc: Hedges’ g =-1.745, 95% CI =-2.513 to -0.977).

*** Indicates a significant difference between the two groups.

P = patient group, HC = healthy control group.

- 1. **Supplementary Tables**

**Supplementary Table 1**. The altered edge numbers of connection within or between modules based on 264 ROIs. The gray shades represent similar results with those of 160 ROIs.

| **Modules** | ***p***  **(FDR Correction)** | **Effect size (Hedges’ g)** | **Edge numbers of connection** |
| --- | --- | --- | --- |
| **Intra-modular Connections** |  |  |  |
| Module 3 (salience, Cingulo-opercular, et al) | 0.005 | -1.009^b^ | ↓ |
| Module 4 (fronto-parietal task control) | 0.001 | -1.195^b^ | ↓ |
| Module 5 (DMN) | 0.001 | -1.198^b^ | ↓ |
| **Inter-modular Connections** | 0.044 |  | ↑ |
| Module 1 (visual) and 5 |  | 0.718^c^ |  |
| Module 2 (sensory/somatomotor) and Module 3 | 0.000 | -1.516^a^ | ↓ |
| Module 3 and 5 | 0.000 | 1.648^a^ | ↑ |

^a, b, c^ Indicate very large, large, and medium effect sizes, respectively
